# Analyzing the effect of public private partnership mode on sewage treatment in China

**DOI:** 10.1038/s41598-024-60055-0

**Published:** 2024-04-25

**Authors:** Xin Wen, Fange Meng, Shiheng Li

**Affiliations:** 1 School of Economics, Capital University of Economics and Business, Beijing, 100070 China; 2 Zhejiang Provincial Branch, The People’s Bank of China, Hangzhou, 310001 China

**Keywords:** Public–private partnership (PPP) mode, Sewage treatment, Government–business relationship, Financial development, Environmental economics, Environmental impact, Socioeconomic scenarios, Sustainability

## Abstract

The public–private partnership (PPP) mode is one of the main ways to promote environmental governance through marketization in the sewage treatment industry. This mode is crucial for environmental protection and livelihood improvement. In order to investigate the impact of PPP mode on sewage treatment, the influence of financial development and the government–business relationship on the effectiveness of sewage treatment under PPP mode, and the role of government in this context, an empirical model is established. To achieve this, data from 284 prefecture-level and above cities in China from 2009 to 2017 has been selected as research samples. The total amount of regional sewage treatment PPP projects is used as the proxy variable for participation in the PPP mode. The findings reveal that the PPP mode of sewage treatment effectively reduces the intensity and amount of sewage discharge. Moreover, the results indicate that a higher level of financial development and a more perfect financial system are associated with better sewage treatment effects under the PPP mode. Similarly, a more harmonious government–business relationship and a higher health index of this relationship correspond to improved sewage treatment effects under the PPP mode. The government should actively enhance government transparency, formulate appropriate corporate taxes and fees, clarify the responsibilities and obligations of the government and enterprises, and optimize the business environment in order to optimize the sewage treatment effect of the PPP mode.

## Introduction

The rapid development of the economy has led to a series of environmental issues that greatly affect our daily lives. As the population grows and the economy expands, the discharge of sewage increases. The United Nations Water Resources Assessment report reveals that globally, millions of tons of waste are deposited into water bodies every day, with wastewater contaminating freshwater sources at a ratio of 1:8. The current sewage treatment capacity is insufficient^1^, necessitating the adoption of new technologies and infrastructure enhancements to boost treatment efficiency^2,3^. The operation of public-owned sewage treatment plants strains government budget^4^. To address funding challenges, governments have turned to public–private partnership (PPP) mode^5,6^, which involve collaboration between the public sector and private investors. The use of the PPP mode aims to alleviate the financial burden on the government and enhance sewage treatment effectiveness^7,8^, albeit sometimes conflicting with profit-maximizing motives of private enterprises. Key issues facing PPP implementation include its efficacy in reducing sewage discharge and the impact of government–business relationship and financial market improvements on sewage treatment outcomes.

Under the current government investment paradigm, the sewage treatment sector faces challenges such as low operational efficiency and lack of market vitality. In addressing these issues, it is imperative for the government to balance economic development considerations alongside environmental protection. Given the financial constraints faced by the government, alternative financing mechanisms are essential. One such approach is the PPP mode, which leverages private capital to deliver public goods and services, fostering mutual benefits and risk-sharing^9^. The World Bank reveals a growing trend in PPP projects within the environmental protection domain, with 1879 projects spanning water and solid waste treatment, amounting to 215.9 billion dollars in investments between 1990 and 2022. Europe emerges as the leader in eco-environment PPP initiatives worldwide, followed by East Asia Pacific and Latin America and the Caribbean. Conversely, Africa and the Middle East region exhibit a relatively low share of such projects. Notably, China has emerged as a frontrunner in implementing PPP projects for ecological and environmental protection, constituting 16.4% of the global total. According to relevant data of the Project Management Library of the Center for Government and Social Capital Cooperation of the Ministry of Finance, as of May 2022, China’s environmental protection projects investment amount ranked fifth, and the number of projects ranked third. China’s commitment is evidenced by notable growth in urban sewage treatment capacity from 160.65 million cubic meters in 2015 to 226.05 million cubic meters in 2022. While these achievements are commendable, there is a need for a comprehensive evaluation of the effectiveness of the PPP mode in sewage treatment projects in China. As the country navigates this critical juncture in advancing environmental protection efforts, a thorough assessment will be pivotal in shaping future strategic directions.

Sewage treatment plants are wastewater treatment facilities. Depending on the treatment approach, there are two types of sewage treatment, centralized and decentralized. Decentralized wastewater treatment treats wastewater directly at the source where it is generated. It is suitable for areas with dispersed populations. These plants have a smaller footprint and lower investment and maintenance costs, making them advantageous for certain scenarios. They can develop different treatment methods to meet specific needs. However, due to their high equipment requirments and small tscale, decentralized systems are not suitable for densely populated cities. Centralized sewage treatment involves collecting regional sewage through a system of pipes and transporting it to a centralized facility for treatment. This method is ideal for areas with concentrated population. Centralized wastewater treatment plants have advanced equipment and operate on a large scale, allowing for efficient monitoring and ensuring that discharge meets standards. The maintenance and operating expenses of centralized wastewater treatment plants are high^10^. In the event of a spill, centralized wastewater treatment plants have a greater impact on the surrounding environment. While centralized plants are costly to maintain and operate, they offer economies of scale and are favored in many developed countries like the UK and Switzerland^11^. In China, where cities are densely populated, centralized wastewater treatment plant have become essential infrastructure in industrial parks since 2015^12^, reflecting the country’s focus on centralized systems.

The PPP mode of collaboration between the public and private sectors has gained popularity as a solution to the limitations of traditional public sector procurement in public infrastructure and services such as water supply, wastewater treament, and environmental protection^13^. Countries likes the United States^14^, New Zealand^15^, the United Kingdom^16^, and Australia^17^, are promoting the adoption of PPP mode. Scholars have conducted extensive research on the effectiveness of the PPP mode, utilizing case analyses, questionnaire surveys, and the difference-in differences (DID) model to evaluate its impact on wastewater treatment. Despite the potential benefits, there have been debates among scholars regarding the efficacy of the PPP mode in controlling water pollution. Some researchers have raised concerns about the PPP mode’s ability to achieve desired outcomes in water pollution treatment. For instance, Henjewele et al. (2014) identified issues such as cost overruns, time delays, and chaning demand patterns in PPP projects based on their analyses^18^. Furthermore, Gong et al. (2019) highlighted the risks associated with incomplete contracts leading to the inefficient implementation of PPP projects and the misallocation of social capital^19^. On the contrary, proponents of the PPP mode argue that it can lead to cost saving and operational efficiencies. Raisbeck et al. (2010) compared traditional projects with PPP project and found that the latter can reduce costs and shorten project duration, especially for larger and more complex endeavors^20^. Similarly, Wang et al. (2020) demonstrated that the PPP mode can address financial needs for project construction and enhance construction efficiency through their questionnaire-based study^21^. The PPP mode p;ays a significant influence in reducing the amount of sewage treatment, according to Hou (2022), who built a DID model and verified the effect of the PPP mode on sewage treatment^22^. However, gaps exist in the existing literature. Studies often rely on case analyses and questionnaire surveys as measurement tools without specific indicators to evaluate the PPP mode. Additionally, the role of the government in supporting and regulating PPP initiatives is often overlooked. Governments play a crucial role in creating a conducive environment for PPP projects by enacting laws, attracting private sector investment, and facilitating PPP development, thereby enhancing the effectiveness of the PPP mode in reducing wastewater discharge. Further research is needed to comprehensively assess whether the PPP mode is indeed effective in reducing sewage discharge and to address these current research limitations.

We conducted a comprehensive analysis of centralized sewage treatment related types of PPP projects in China by manually collating data from the project management database of the Ministry of Finance and the Social Capital Cooperation Center. Specifically, the total amount of regional sewage treatment PPP projects was used as a proxy variable to measure participation in the PPP mode, assessing the effectiveness of centralized sewage treatment in 284 Chinese cities from 2009 to 2017. The study focused on evaluating the influence of financial development and government–business relationship on the effectiveness of sewage treatment, as well as the role of the government in environmental governance. The findings indicate that the PPP mode can significantly reduce sewage discharge, particularly when coupled with improvements in the financial market and business environment. We introduces two key contributions to the existing literature. Firstly, it employs specific indicators to gauge participation in the PPP mode, which differs from the conventional approach of using questionnaires or case studies. By utilizing the total amount of regional sewage treatment PPP projects as a proxy variable, this research enables direct comparisons across time and space while shedding light on the involvement of social capital and delineating the roles of government and business in the PPP mode. Secondly, it explores how financial development and government–business relationship impact the effectiveness of sewage treatment in the PPP mode, underscoring the governmental role in this domain. Unlike previous studies that mainly focus on the efficacy of sewage treatment under the PPP mode, this research highlights the significance of the financial and market environment in influencing the participation of social capital in providing public goods. Thus, this study enriches the existing literature on PPP mechanisms and offers valuable insights for both developed and developing countries in enhancing the design and implementation of such mechanisms.

The subsequent sections of the paper are structured as follows: a literature review and institutional background constitute the second part, followed by research design in the third section. The empirical tests in the fourth part delve into the analysis of the impact of PPP mode on sewage discharge intensity, while the fifth part explores how financial development and government–business relationship influence the efficacy of sewage discharge under the PPP mode. The paper concludes with the sixth part, encompassing research findings and policy recommendations.

## Literature review and institutional background

### Literature review

The PPP mode, first proposed by the UK in the 1960s and 1970s, emerged with a clear terminology and framework. This model aims to introduce social capital by utilizing a principal-agent approach to enhance resource allocation efficiency and share risks between the public and private sectors^23^. In recent years, governments in European countries have increasingly adopted the PPP mode for co-financing and developing public infrastructure^24,25^. Furthermore, as human production and living activities continue to give rise to environmental issues, the PPP mode has been utilized in environmental protection initiatives^26^, particularly in addressing water pollution and implementing renewable energy projects^27^.

The effectiveness of the PPP mode in enhancing sewage treatment efficiency is a topic that remains to be empirically validated. Scholars hold divergent views on the matter, with some asserting the positive impact of the PPP mode on urban sewage treatment efficiency. They argue that PPP projects can enhance financial capacity, mitigate government debt risks, elevate project management and operations, as well as drive innovation and optimize management strategies^28^. By engaging the private sector, there is potential to address issues related to poor service quality resulting from government monopolies^29^ and enhance operational efficiency within the public sector^30^. The collaboration between the public and private sectors under the PPP model has the potential to minimize resource wastage, lower production costs, combat water pollution, and reduce government subsidies^31–33^. Additionally, it can enhance allocative efficiency^34^ and foster innovation by introducing competitive market dynamics^35^. Recent research has contributed valuable insights into the environmental performance of the PPP mode in urban sewage treatment. For instance, Tang et al. (2021) examined enterprise data from Jiangsu province, China, revealing that the PPP mode led to enhance pollutant treatment performance through increased operational costs and improved sewage treatment efficiency^36^. Moreover, Hou (2022) leveraged a difference-in-differences (DID) model and panel data from 267 prefecture-level cities in China demonstrate the positive impact of the PPP mode and national demonstrative characteristics on sewage treatment capacity^22^. Despite these findings, some scholars express reservations regarding the efficacy of the PPP mode in sewage treatment. They argue that while it may alleviate funding shortages, conflicts may arise between overarching environmental goals and individual profit motives. Critics highlight concerns that private sector involvement in PPP projects, primarily driven by economic incentives, could compromise environmental conservation efforts. Doubts also linger regarding the quality of services delivered by private providers focused on profit margins^37,38^. In developing countries, the inherent economic and financial risks stemming from regulatory loopholes and institutional shortcomings can challenge the government's ability to attract social capital^39,40^. Moreover, the implementation of the PPP mode introduces uncertainties like transaction costs, financing challenges, and potential cost overruns^41,42^.

Hypothesis 1.1: the PPP mode can improve the efficiency of sewage treatment and reduce the intensity of sewage discharge.

Hypothesis 1.2: the PPP mode cannot improve the efficiency of sewage treatment.

The sewage treatment industry is closely tied to both production and daily life, with a relatively inelastic demand. The government’s financial allocation towards sewage treatment projects remains limited. Relying solely on the government for investments in the sewage treatment sector could potentially strain the government’s financial resources and lead to significant liabilities. In cases where government expenditures fall short of meeting the demands for sewage treatment, the resulting investment gap may result in an oversupply of demand. In addressing this challenge, the public–private partnership (PPP) model emerges as a novel financing approach distinguished by private sector involvement in public ventures. Through the PPP model, various avenues of investment can be extended to the sewage treatment industry. It is essential to note that the form and method of financing play a pivotal role in the ultimate execution of projects^43^. Financing intricacies pose significant hurdles for the PPP mode’s implementation in the current landscape^44^. A robust financial market offers a range of financing mechanisms for PPP projects, mitigating risks associated with concentrated financing. Additionally, it enhances the distribution of returns among stakeholders, while facilitating the entry and exit strategies for social capital. A well-functioning financial market ecosystem can effectively stimulate private sector interest in financing ventures, with the market’s maturity serving as a critical determinant of PPP project financing success^45^.

Hypothesis 2: other conditions being equal, regional financial development affects the effect of PPP projects.

In the process of operation, the government needs to adjust the relationship between the government and the private sector. There are some problems in the PPP mode, such as the asymmetry of responsibilities and rights, outdated management methods, and the lack of management talents in the market economy environment. Literature has shown that the financial capacity of governments affects the participation of the private sector in PPP projects^46^. In the actual implementation of PPP projects, there are still many problems such as irregular contracts, disguised outsourcing of public functions, and social capital bearing the “burden of public goods”^19^. Therefore, the government needs to clarify its regulatory status and power boundaries. When the public sector can clarify its power boundaries, it can reduce the burden of public goods on the private sector, and the PPP mode can become a long-term channel for public goods supply^19^. A “clear” and “pro” government–business relationship can reduce the information asymmetry between enterprises and financial institutions and enable enterprises to obtain loans^47^. Simultaneously, the government improves the transparency of information exchange with enterprises and reduces the government’s intervention in enterprises, which can promote the improvement of innovation capacity^48^. The healthier the government–business relationship, the more conducive it is to eliminating problems such as information asymmetry and lax regulation, which is conducive to enhancing the effect of sewage treatment in the PPP mode.

Hypothesis 3: other conditions being equal, the government–business relationship affects the effect of PPP projects.

### Institutional background

Since the 1990s, the PPP mode has gained popularity in the field of public infrastructure in western countries, especially in Europe, as a novel financing method^49^. This mode involves the government granting long-term concessions and revenue rights to the private sector, enabling the acceleration of infrastructure construction and its efficient operation. By allowing more social capital to get involved in public infrastructure projects, the PPP mode eases the government’s initial investment burden, reduces risk, and enhances service quality. Social capital stands to benefit financially under this mode. Various countries have explored the potential of the PPP mode, with the United Kingdom primarily utilizing concessions and private finance initiatives (PFI) in infrastructure projects^50^. In France, local governments oversee the drinking water and sewage treatment sectors, with private companies managing sewage treatment plants in a competitive manner alongside the public sector^51^. Scholars have also investigated the application of the PPP mode in developing countries, with studies like Jesintha’s research in 2011 indicating that public–private partnership have driven progress in India^52^. Facing inefficient and underfunded sewage treatment in the mid-1990s, China embarked on applying the PPP mode in the sewage treatment industry to balance treatment needs with economic growth. In 1995, China introduced the “Guidelines for Pilot Establishment of a Modern Enterprise System for Municipal Public Enterprises,” initiating the opening up of the sewage treatment sector’s monopoly. Despite improvements in the capital market, institutional barriers such as laws and regulations persisted. By 2004, the government endorsed market-oriented reforms for public utilities, with the implementation of the “municipal public utilities franchise management approach” urther spurring the franchising of the sewage industry as a market access mechanism. Subsequently, the country witnessed the swift adoption of build-operate-transfer (BOT) and transfer-operate-transfer (TOT) models for franchising, expediting the marketization of the sewage sector. The policy frameworks outlined in the “Twelfth Five-Year Plan” and the “Thirteenth Five-Year Plan” provided essential support for the advancement of the sewage treatment industry. Meanwhile, through prudent price regulation, the government ensured that some of the economic gains from social capital’s participation in the sewage sector were passed on to consumers, thereby enhancing overall social welfare.

The emergence of the PPP mode in China’s sewage treatment industry has gradually made the market more oriented due to the evident contradiction between the strong demand for machinery and equipment in the sector, and the limited supply of funds leading to low treatment efficiency^53^. To ensure the successful implementation of the PPP mode, the government has introduced a series of laws and regulations. The effectiveness of the PPP model in reducing sewage discharge and the extent to which government intervention influences its role are areas that require further verification.

## Research design

### The sewage treatment effect model setting of PPP mode

The following measurement model is established to test the effect of the public–private partnership (PPP) mode on sewage discharge intensity. Model I, referred to as the impact model, serves as a basic regression analysis that examines the overall influence of the government’ adoption of the PPP mode on sewage discharge intensity. Model II focuses on the moderating effect and delves into whether factors such as financial development and government–business relationship impact the relationship between PPP mode and sewage discharge intensity.

Model I: effect model of PPP mode on the intensity of sewage discharge.

1 $$wate{r_{it}}{ = }{\alpha_0} + {\alpha_1}PP{P_{it}} + \alpha {X_{it}} + {\theta_i} + {\varepsilon_{it}} $$

Model II: moderating effect model.

2 $$ wate{r_{it}}{ = }{\beta_0} + {\beta_1}PP{P_{it}} + {\beta_2}{Z_{it}} + {\beta_2}PP{P_{it}} \times {Z_{it}} + {\beta_3}fi{n_{it}} + \beta {X_{it}} + {\varepsilon_{it}} $$

In Models I and II, the basis for evaluating the impact of the public–private partnership (PPP) mode on sewage discharge lies in the estimated coefficient of the dependent variable, sewage discharge intensity (*water*), and the independent variable, the total amount of the PPP project (*PPP*). To further investigate the influence of financial development and government–business relationship on the government’s PPP mode with respect to sewage discharge, financial development (*fin*) and government–business relationship (*hpbr*) are introduced as moderating variables (*Z*_*it*_) in Model II. Additionally, control variables (*X*_*it*_) encompass industrial structure (*structure*), total industrial output (*output*), industrial size (*size*), financial capacity (*finance*), and economic growth (*growth*).

### Variable selection

The independent variable, PPP mode (PPP) measures the sewage treatment PPP mode with the total amount of sewage treatment PPP projects in the region in the current year. The reason for using the sewage discharge per unit output value as the dependent variable, sewage discharge intensity (*water*), rather than the amount of sewage discharge is that the unit sewage discharge is more comparable in terms of different production scales. The moderating variable financial development (*fin*) is determined by the ratio of financial institutions’ loan balances to GDP. The government–business relationship health index, a moderating variable denoted as *hpbr*, accesses the relationship between the government and enterprises. A higher index signifies a healthier government–business relationship.

To improve the accuracy of the estimation by addressing missing variables, the regression model includes several control variables. First, the industrial structure (*structure*) is incorporated, which is defined by the proportion of the output value of the secondary industry in the total output value. The secondary industry includes manufacturing, mining, and energy sectors known for high pollution levels. A higher proportion of the secondary industry implies increased resource consumption and sewage discharge^54^. Secondly, the total industrial output value (*outpt*) is included, indicating the city’s industrial production scale for the year. The industrial scale (*size*) parameter features the number of industrial enterprises in the city during that year. A greater number of industrial enterprises lead to a more dispersed distribution, weaker government supervision, and higher sewage discharge intensity. Furthermore, financial capacity (*finance*) is taken into account, measured by the ratio of general budget revenue to general budget expenditure. A higher ratio signifies stronger regional financial capacity, enhanced financial support for environmental protection, and reduced sewage discharge intensity. Lastly, economic growth (*growth*) is considered, as increased growth may prompt authorities to relax environmental protection requirements to enhance sewage discharge intensity.

### Sample selection and data source

The study on “Promoting the Integration of Large-scale Resettlement Areas for Poverty Reduction and Relocation into New Urbanization to Achieve High-Quality Development” highlights the significant migration of China’s rural populace to urban centers. With China’s rapid urbanization, cities have become densely populated. To accommodate this concentration of population, centralized sewage treatment systems have been implemented. In our investigation, we focused on the utilization of public–private partnership (PPP) projects in sewage treatment to assess the adoption of centralized sewage treatment. To conduct our analysis, data from Chinese prefecture-level and above cities between 2009 and 2017 were utilized, resulting in a study sample of 284 cities after the exclusion of locations with missing data. The omitted cities include Laiwu in Shandong, Bijie, and Tongren in Guizhou, as well as Sansha, Danzhou in Hainan, Shizuishan in Ningxia, Haidong in Qinghai, Shigatse, Changdu, Linzhi, Shannan and Naqu in Xizang, and Turpan and Hami in Xinjiang. The final sample comprised 2556 observed values over a span of 9 years.

Missing individual data for specific years and cities is classified as missing values in this study. The data for the total amount of sewage treatment PPP projects in the primary explanatory variable area is collected manually by the Government and Social Capital Cooperation Center of the Ministry of Finance, serving as one of the main data sources. Additionally, data on sewage discharge, industrial structure, total industrial output, industrial size, financial capacity, economic growth, and financial development are sourced from the Statistical Yearbook of Chinese Cities. Any individual data missing for specific years or cities is handled as a missing value in the analysis.

## Regression results

### Descriptive statistics

Table 1 shows the descriptive statistics of the main variables. The average total amount of PPP sewage treatment projects in 284 cities in China is 315 million yuan. The maximum value of 32 billion yuan was recorded in Beijing in 2017, while the lowest value of 0 yuan was predominantly observed in cities before 2015.

**Table 1:** Descriptive statistics of variables.

Variable	Unit	Sample size	Max	Min	Mean	Standard deviation
*water*	tons/ten thosand yuan	2556	251.177	0.114	3.942	7.836
*PPP*	billion yuan	2556	320	0	3.15	12.666
*structure*	%	2556	89.75	13.57	48.813	10.524
*output*	billion yuan	2556	32,000	11.418	3282.262	4445.155
*size*	each	2556	17,906	20	1308.572	1688.877
*finance*	%	2556	31.852	0.647	2.767	1.956
*growth*	%	2556	109	− 19.380	10.289	4.579
*fin*	%	2556	7.450	0.068	0.889	0.562
*hpbr*		2556	1	0	0.281	0.152

### The regression results of sewage treatment effect of PPP mode

Using the balance panel data of 284 cities in China at prefecture level and above from 2009 to 2017 as samples, Model I is employed to empirically test the impact of the sewage treatment PPP project on sewage discharge. The total amount of the sewage treatment PPP project serves as the core explanatory variable, while sewage discharge is the dependent variable. The findings of this analysis are presented in column (1) of Table 2.

**Table 2:** Basic regression of water pollution discharge affected by sewage treatment PPP project.

	Water				
	(1)	(2)	(3)	(4)	(5)
PPP	− 0.030***	− 0.252***	− 0.032***	− 0.282***	− 0.030***
	(− 9.83)	(− 9.34)	(− 9.73)	(− 8.11)	(− 10.36)
Structure	0.007*	0.008**	0.012***	0.013***	0.007**
	(1.84)	(2.17)	(3.08)	(3.31)	(2.11)
Output	− 1.024***	− 1.033***	− 1.114***	− 1.123***	− 0.997***
	(− 24.13)	(− 23.83)	(− 21.00)	(− 20.68)	(− 23.61)
Size	− 0.093	− 0.083	− 0.061	− 0.057	− 0.088
	(− 1.35)	(− 1.18)	(− 0.88)	(− 0.81)	(− 1.32)
Finance	− 0.020	− 0.020	− 0.033*	− 0.033*	− 0.014
	(− 1.43)	(− 1.45)	(− 1.83)	(− 1.83)	(− 0.98)
Growth	0.011***	0.011***	0.011**	0.011**	0.010***
	(2.94)	(2.65)	(2.28)	(2.30)	(2.94)
Urban characteristics	Controlled	Controlled	Controlled	Controlled	Controlled
R^2^	0.609	0.611	0.557	0.552	0.613
F	225.88	220.85	185.20	174.35	225.85
Sample size	2556	2556	2272	2272	2556

The coefficient is the standardized coefficient. The T value based on the robustness standard error is in brackets, and the estimation result of the constant term is omitted.

*, **, *** indicate that the estimation coefficient is significant at the level of 0.1, 0.05 and 0.01.

The estimated coefficient of the total amount of sewage treatment PPP projects in column (1) of Table 2 shows a significant negative relationship. This implies that the adoption of the PPP mode leads to a notable reduction in the intensity of sewage discharge, highlighting the effectiveness of implementing PPP projects in reducing sewage levels. By utilizing social capital financing, the PPP mode addresses the funding shortage issue in the sewage treatment industry and diversifies financial risks^23^. The infusion of social capital also enhances the competition mechanism, thereby fostering better resource allocation efficiency and improving sewage treatment effectiveness. Consistent findings are obtained with previous studies by Hou (2022)^22^ and Tang et al. (2021)^36^. Hou (2022) utilized the DID model to examine the sewage treatment outcomes of the PPP model. Although DID is an effective method for evaluating policy impacts, there may be challenges related to the exogeneity of the experimental group selection. On the other hand, Tang et al. (2021) focused solely on data from Jiangsu province to analyze the effects of PPP mode on sewage treatment, potentially limiting the generalizability of their results. We use the total amount of sewage treatment PPP projects as a proxy variable for participation in PPP initiatives, thus circumventing issues related to experimental group selection and directly reflecting the extent of PPP project participation. Regardless of whether DID or proxy variables are employed, our results consistently demonstrate that the PPP model enhances sewage reduction effects, thereby confirming hypothesis 1.1.

The estimated coefficient of industrial structure is significantly positive from the results of the control variables, suggesting that a higher proportion of the secondary industry leads to increased intensity of sewage discharge. This is primarily due to the rapid growth of high energy consumption and high pollution industries like manufacturing and mining in areas with a higher share of secondary industries. This growth results in a greater demand for sewage discharge, thus increasing the overall intensity of sewage discharge. On the other hand, the estimated coefficient of the total industrial output value is significantly negative at the 1% significance level, indicating that higher total industrial output values are associated with better sewage treatment effects. As total industrial output values increase, so does the industrial development level. The regression coefficient of economic growth is significantly positive, showcasing that regions with higher economic growth experience higher sewage discharge intensities. In efforts to drive economic development, local governments often lower entry barriers and relax environmental regulations for enterprises, consequently raising regional sewage discharge intensities. Meanwhile, the estimation coefficient of government financial capacity is negative but not statistically significant.

### Robustness test

In order to ensure the robustness of the results, we utilized both the replacement variable method and the lagged variable method for conducting additional testing. The replacement variable method involved using the number of sewage treatment PPP projects as a metric to gauge the extent of the adoption of the PPP mode in regional sewage treatment endeavors. A higher number of regional sewage treatment PPP projects were indicative of stronger support for sewage treatment embracing the PPP mode. The outcomes obtained from the replacement variable method can be found in column (2) of Table 2. Conversely, the lagged variable method accounted for the time lag effect of PPP projects. This method involved examining the total quantity of sewage treatment PPP projects as well as the number of projects from the previous period to ascertain their impact. The results from these analyses are displayed in columns (3) and (4) of Table 2. In the regression analyses conducted across columns (2), (3), and (4) of Table 2, it was found that the estimated coefficients for the number of sewage treatment PPP projects, the cumulative number of lagged sewage treatment PPP projects, and the project count all displayed significant negative values. Furthermore, the adverse impact of the adoption of the PPP mode in sewage treatment on sewage discharge intensity remained consistent even after the substitution of variables and the introduction of a lag period. The results depicted in column (1) of Table 2 were validated, upholding the anticipated outcomes. This affirmed conclusion demonstrates a consistency with the expected results, highlighting the noteworthy influence of the PPP mode in diminishing sewage discharge. The implication drawn from this is that local governments should intensify their endeavors to attract private investment in order to actualize a mutually beneficial scenario.

After conducting the shrinking-tail test, the results are shown in column (5) of Table 2 to verify the robustness of the conclusions, considering the large gap between the maximum and minimum values of the data, as well as the presence of extreme values. Following this test, it becomes evident that the estimated coefficient of the total amount of sewage treatment PPP projects is significantly negative, further reinforcing the robustness of the findings.

### Heterogeneity test

The city’s resource endowment and market environment are diverse, as are the dominant sectors. The government has imposed strict environmental regulation to varied degrees in an effort to boost the economy. China’s 11th Five-Year Plan for Environmental Protection classifies cities into environmental priority cities and non-environmental priority cities based on varying degrees of environmental regulation. Environmental priority cities are subjected to stricter environmental standards compared to non-environmental priority cities. The geographical division of cities along the “Qinling–Huaihe” line into northern and southern regions brings about distinctions in the business environment and cultural norms between the two regions. Notably, columns (1) and (2) of Table 3 analyze the impact of implementing the PPP mode on sewage discharge in environmental priority cities and non-environmental priority cities, while columns (3) and (4) assess its effects in the southern and the northern regions. The findings in Table 3 reveal a significant decrease in sewage discharge intensity following the implementation of the PPP mode in both environmental priority and non-environmental priority cities, as well as in the southern and northern regions. Moreover, the robustness of the regression analysis in Table 2 underscores the effectiveness of the PPP mode in significantly reducing sewage discharge levels acorss cities with diverse environmental regulations and cultural practices.

**Table 3:** Heterogeneity test.

	Water			
	(1)	(2)	(3)	(4)
	Environmental priority cities	Non-environmental priority cities	Southern cities	Northern cities
PPP	− 0.024***	− 0.037***	− 0.025***	− 0.034***
	(− 6.64)	(− 8.14)	(− 6.83)	(− 6.70)
Structure	0.007	0.008	0.005	0.008
	(1.62)	(1.54)	(0.89)	(1.60)
Output	− 0.974***	− 1.036***	− 1.109***	− 0.944***
	(− 18.46)	(− 17.84)	(− 18.73)	(− 14.61)
Size	− 0.034	− 0.101	− 0.150	− 0.090
	(− 0.46)	(− 0.97)	(− 1.57)	(− 0.95)
Finance	− 0.020*	− 0.020	0.024	− 0.039***
	(− 1.67)	(− 1.16)	(0.84)	(− 4.01)
Growth	0.005*	0.014***	0.013*	0.007*
	(1.80)	(2.90)	(1.72)	(1.73)
Urban characteristics	Controlled	Controlled	Controlled	Controlled
R^2^	0.617	0.615	0.686	0.515
F	92.68	158.65	150.10	77.64
Sample size	1071	1485	1377	1179

The coefficient is the standardized coefficient. The T value based on the robustness standard error is in brackets, and the estimation result of the constant term is omitted.

*, **, *** indicate that the estimation coefficient is significant at the level of 0.1, 0.05 and 0.01.

## Moderating effects of financial development and government-business relationship

The financial market regulatory mechanism has been continuously improved in the process of progressive reform of the financial system, leading to increased support for small and medium-sized enterprises and an overall maturation of the financial system. However, significant regional disparities persist in the level of financial development. A mature financial system enhances the ability of financial services to support the real economy, provides wider financing channels, and facilitates easier access to financial support for enterprises, thereby alleviating their financing constraints. This study employs Model II to investigate whether there are variations in the impact of implementing the PPP mode on the intensity of sewage discharge reduction based on the level offinancial development. The regression results displayed in column (1) of Table 4 reveal a significantly negative regression coefficient for the cross-multiplier term (PPP*Z), indicating that financial development amplifies the inhibitory effect of the PPP mode on effluent discharge intensity. These findings confirm hypothesis 2, suggesting that the PPP mode is more effective in reducing pollution emissions in regions with higher financial development levels due to expanded financing channels, strengthened financial market regulatory mechanisms, and improved access to capital for small and medium-sized enterprises^43^.

**Table 4:** Moderating test.

	Water			
	(1)	(2)	(3)	(4)
PPP	−0.001	−0.0001	0.002**	−0.001
	(−0.42)	(−0.10)	(1.98)	(−0.37)
Z	−0.032	1.718***	0.249***	0.017***
	(−0.57)	(5.69)	(3.55)	(4.14)
PPP*Z	−0.016***	−0.065***	−0.009***	−0.001***
	(−4.33)	(−6.62)	(−10.47)	(−5.63)
Structure	0.007**	0.009***	0.005	0.009***
	(2.20)	(2.84)	(1.56)	(2.85)
Output	−0.983***	−0.995***	−0.934***	−0.998***
	(−23.60)	(−24.00)	(−23.02)	(−23.87)
Size	0.346***	0.289***	0.308***	0.309***
	(6.80)	(5.33)	(6.23)	(5.52)
Finance	−0.040***	−0.036***	−0.032**	−0.037***
	(−2.90)	(−2.65)	(−2.44)	(−2.67)
Growth	0.013***	0.013***	0.011***	0.013***
	(2.87)	(3.03)	(2.99)	(3.02)
Urban characteristics	Controlled	Controlled	Controlled	Controlled
R^2^	0.564	0.571	0.584	0.567
Sample size	2556	2556	2556	2556

The coefficient is the standardized coefficient. The T value based on the robustness standard error is in brackets, and the estimation result of the constant term is omitted. Z represents the moderating variable, which are the health index, closeness index and cleanliness index of government–business relationship.

*, **, *** indicate that the estimation coefficient is significant at the level of 0.1, 0.05 and 0.01.

The impact of the government–business relationship on the effectiveness of the PPP mode in mitigating sewage discharge levels is evaluated through various indices—namely, the government–business relationship health index, innocence index, and closeness index. These indices are analyzed in columns (2), (3), and (4) of Table 4 to determine their relationship with the cross-multiplier term (PPP*Z) representing the total amount of sewage treatment PPP projects. The regression coefficients for the health index, innocence index, and closeness index displayed in Table 4 indicate a significant negative correlation with the cross-multiplier term, suggesting that a strong government–business relationship enhances the PPP mode’s ability to reduce sewage discharge intensity. These findings confirm the validity of hypothesis 3, which asserts that a positive government–business relationship contributes to operational efficiency. By fostering effective collaboration between the government and enterprises, integrating market dynamics with government initiatives, and enhancing the health index of the government–business relationship, the regulatory environment can be optimized. Creating a conducive business environment involves curbing excessive government intervention in sewage treatment ventures, defining clear roles and responsibilities for both parties, and streamlining operational efficiency. A higher innocence index signifies enhanced governmental transparency and integrity thereby bolstering public trust, curbing misuse of authority, and preventing deviations implementation. Moreover, a higher closeness index denotes improved government support for enterprises, rationalized tax structures, reduced operational costs for enterprises engaged in sewage treatment PPP projects, and increased profitability. Such conducive conditions attract more enterprises to partake in sewage treatment PPP projects, ultimately leading to a reduction in sewage discharge intensity.

## Conclusion and policy recommendations

This study utilizes the data from 284 prefecture-level and above cities in China between 2009 and 2017 as research samples. The total number of regional sewage treatment PPP projects serves as the proxy variable for assessing participation in the PPP mode. The primary objective is to assess the influence of the PPP mode on sewage discharge intensity and examine how financial development and government–business relationship may affect its efficacy in sewage treatment. The findings reveal that the PPP mode effectively reduces sewage discharge intensity by fostering collaboration between the government and social capital. This collaborative approach contributes to diminishing sewage discharge levels and achieving environmental management goals. Importantly, these results are confirmed through robustness tests employing alternative variables and lagged explanatory variables. Furthermore, the moderating effect analysis indicates that the impact of the PPP mode in decreasing sewage discharge intensity is enhanced in environments characterized by higher levels of financial development, harmonious government–business relationship, and a healthier the government–business relationship index. In light of these empirical findings, this study proposes the following policy recommendations:

In the field of infrastructure construction, attention should be given to the introduction of social capital to ease the pressure on government infrastructure development and to enhance urban infrastructure projects. A study of the PPP mode in sewage treatment has revealed that this approach can effectively reduce sewage discharge. While acknowledging the positive impact of the existing PPP mode, it is essential to prioritize the quality of social capital during its introduction. To facilitate the entry of private entities into the sewage treatment sector, the government must conduct thorough screenings. This process should consider not only the financial capacity of the social capital being introduced but also evaluate the level of expertise in sewage treatment. Furthermore, the government should leverage verage the expertise and operational strategies of leading the efficiency and quality of sewage treatment. Establishing clear boundaries of authority is crucial to ensuring that the PPP mode can serve as a sustainable source for the sewage treatment industry in the long term.

To enhance the role of public–private partnership (PPP) in sewage treatment and foster a conducive business environment, several key measures should be taken by the government. Firstly, the government needs to strengthen its communication with the business sector and improve the capacity and quality of services provided. Furthermore, setting reasonable taxes and fees as well as optimizing the business environment are crucial steps to be undertaken. This will contribute to elevating the health index of the government–business relationship, which in turn will boost the effectiveness of PPP in sewage treatment projects, particularly in regions with favorable business environments. In managing the inherent conflicts of interest between individual profit motives and societal benefit, a delicate balance needs to be struck. Specifically, under the PPP mode, private sector entities typically prioritize profit maximization. To ensure the optimal reduction in sewage discharge, the government must exercise flexible macro-control. In instances where project returns are low, appropriate subsidies and incentives should be provided by the government to incentivize private sector participation. This strategy aligns profit maximization with environmental protection goals, thus promoting the sustainable development of sewage treatment projects within the framework of PPP.

To enhance the effectiveness of sewage treatment, several strategies can be implemented. Firstly, technical equipment updates play a crucial role in improving sewage treatment. By upgrading the technical equipment, the efficiency of sewage treatment can be significantly enhanced. Secondly, improving the financial market is essential in expanding financing channels for enterprises involved in PPP projects. Private enterprises can obtain financing loans, which not only solves their financial issues but also promotes technological research and development (R&D) and innovation. This, in turn, facilitates technological updates that contribute to enhancing sewage treatment efficiency. Moreover, utilizing digital platforms within the financial sector can help bridge the information gap between financial institutions and enterprises, enabling them to access a wider range of financing options. By integrating these strategies, the overall sewage treatment process can be more efficient and sustainable.

This study, based on existing data, can only assess the short- and medium-term implementation effects of the sewage treatment PPP project. However, there is a lack of experimental evidence regarding the long-term implementation effects of the policy. In the future, the promotion of PPP projects will require real-time data analysis for dynamic adjustments to achieve optimal results.

## Data Availability

The datasets generated during and/or analysed during the current study are available from the corresponding author on reasonable request.
